# The genome sequence of Natterer’s Bat, *Myotis nattereri* (Kuhl, 1817) (Chiroptera: Vespertilionidae)

**DOI:** 10.12688/wellcomeopenres.27171.1

**Published:** 2026-07-25

**Authors:** Matthieu Ménage, Gwendoline Duménil, Myrtani Pieri, Sonja C. Vernes, Emma C. Teeling, Meike Mai, Wellcome Sanger Institute Tree of Life Management, Samples and Laboratory team, Wellcome Sanger Institute Scientific Operations: Sequencing Operations, Wellcome Sanger Institute Tree of Life Core Informatics team, Tree of Life Core Informatics collective, Darwin Tree of Life Consortium

**Affiliations:** 1 CAWA, Plouër-sur-Rance, Brittany, France; 2 Department of Life Sciences, School of Life and Health Sciences, University of Nicosia, Nicosia, Cyprus; 3 School of Biology, University of St Andrews, St Andrews, Scotland, UK; 4 University College Dublin School of Biology and Environmental Science, Dublin, Leinster, Ireland; 5 Tree of Life Programme, Wellcome Sanger Institute, Hinxton, England, UK

**Keywords:** Myotis nattereri, Natterer’s Bat, genome sequence, chromosomal, Chiroptera

## Abstract

We present a genome assembly from an individual male *Myotis nattereri* (Natterer’s Bat; Chordata; Mammalia; Chiroptera; Vespertilionidae). The assembly contains two haplotypes with total lengths of 2 138.58 megabases and 1 932.61 megabases. Most of haplotype 1 (98.98%) is scaffolded into 23 chromosomal pseudomolecules, including the X and Y sex chromosomes. Haplotype 2 was assembled to scaffold level. The mitochondrial genome has also been assembled, with a length of 17.27 kilobases. Gene annotation of this assembly on Ensembl identified 19 245 protein-coding genes. This assembly was generated as part of the Darwin Tree of Life project, which produces genomes for eukaryotic species found in Britain and Ireland.

## Species taxonomy

Eukaryota; Opisthokonta; Metazoa; Eumetazoa; Bilateria; Deuterostomia; Chordata; Craniata; Vertebrata; Gnathostomata; Teleostomi; Euteleostomi; Sarcopterygii; Dipnotetrapodomorpha; Tetrapoda; Amniota; Mammalia; Theria; Eutheria; Boreoeutheria; Laurasiatheria; Chiroptera; Yangochiroptera; Vespertilionidae; *Myotis*; *Myotis nattereri* (Kuhl, 1817) (NCBI:txid109481).

## Background

*Myotis nattereri* (Natterer’s bat) is a medium-sized vespertilionid bat belonging to the genus *Myotis*, a species-rich mammalian genus with over 120 extant species distributed globally ( Gunnell *et al.*, 2017). Molecular phylogenetic studies have revealed that *M. nattereri* represents a cryptic species complex comprising at least four well-differentiated lineages in the Western Palaearctic, demonstrating that morphological similarity can mask substantial genetic diversity ( Salicini *et al.*, 2011). The genus *Myotis* has an ancient evolutionary history extending over 33 million years to the early Oligocene, with East Asia identified as the likely cradle for its cosmopolitan radiation ( Gunnell *et al.*, 2017; Ruedi *et al.*, 2013). Within Vespertilionidae, *Myotis* forms a monophyletic group supported by both mitochondrial and nuclear markers, though ecomorphological convergence has resulted in similar phenotypes evolving independently across geographic regions ( Ruedi & Mayer, 2001).

**Figure 1.**
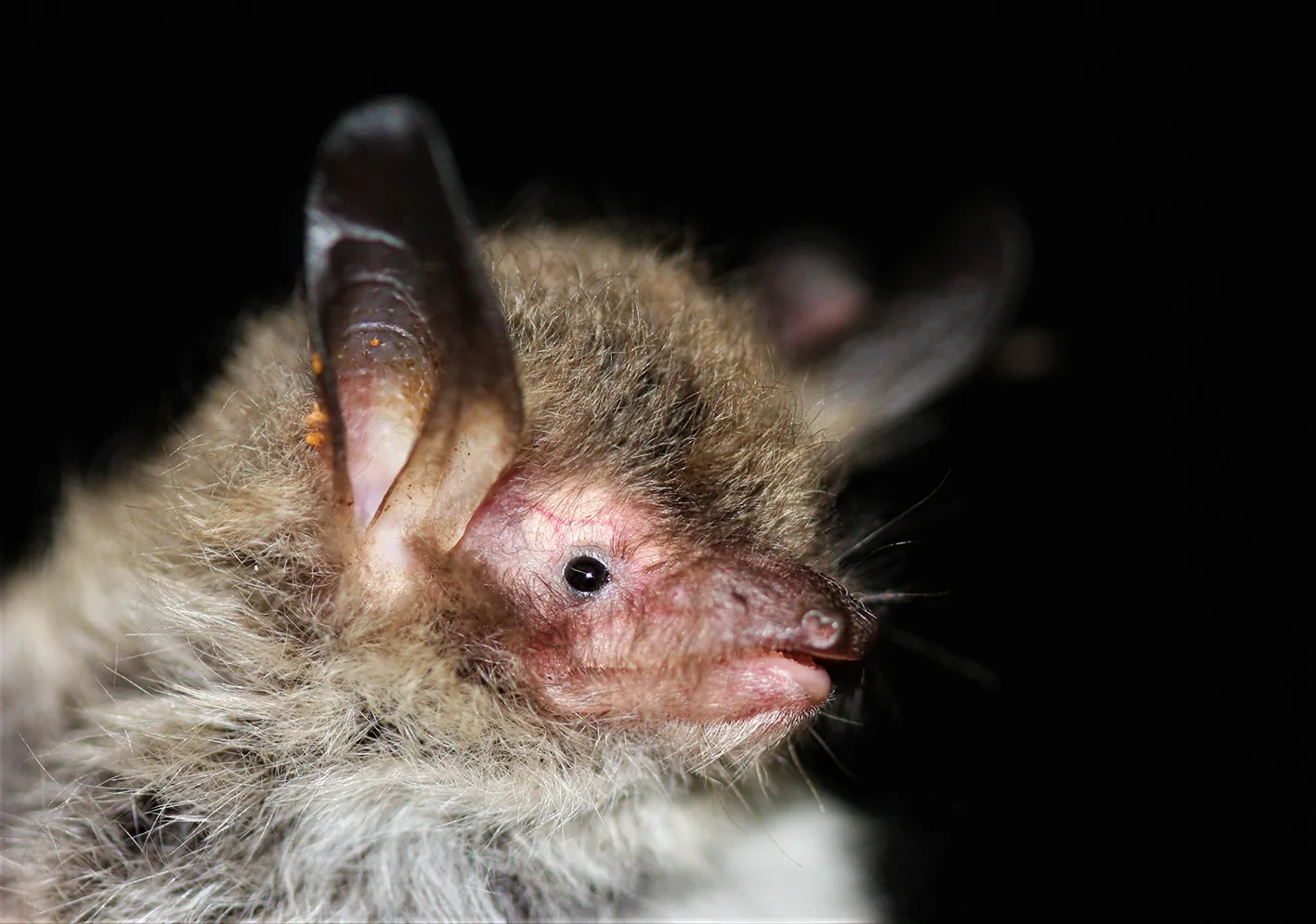
Photograph of *Myotis nattereri* by Matthieu Ménage (Brittany, France).

*M. nattereri* ( Figure 1) is widely distributed across Europe, as documented in field studies from England, Germany, and other European countries ( August *et al.*, 2014; Hope *et al.*, 2014; Rivers *et al.*, 2005). The species inhabits woodland and forested landscapes, where it forms stable, mixed-sex social groups with exclusive roost home ranges averaging 0.2 km ^2^ ( August *et al.*, 2014). Roosting occurs primarily in buildings, including historic churches where maternity colonies can reach considerable size ( Zeale *et al.*, 2016). *M. nattereri* exhibits a flexible foraging strategy, employing both aerial hawking and gleaning to capture prey ( Melcón *et al.*, 2007). Dietary analyses using molecular methods have revealed an unexpectedly broad diet that includes substantial proportions of non-volant prey such as lepidopteran larvae, spiders, and other ground-dwelling arthropods, with foraging activity documented even during winter in mild climates ( Hope *et al.*, 2014). This gleaning behaviour is facilitated by specialised echolocation calls with relatively high bandwidth that enable detection of prey in cluttered environments ( Siemers & Schnitzler, 2004).

Reproductive biology in *M. nattereri* is characterised by delayed sexual maturity, with only 12.85% of males reaching reproductive condition as juveniles, compared to higher rates in sympatric congeners ( Linton & Macdonald, 2020). Mating occurs primarily at autumn swarming sites near hibernacula, where large numbers of bats from different summer colonies congregate, facilitating gene flow and maintaining high genetic diversity despite female philopatry ( Rivers *et al.*, 2005). Spring weather conditions strongly influence reproductive success, particularly in first-year females, with precipitation during implantation or early pregnancy significantly reducing breeding success ( Linton & Macdonald, 2018; Stapelfeldt *et al.*, 2022). Parturition timing and juvenile development are sensitive to climatic variation, and reproductive rates increase with female age ( Linton & Macdonald, 2018). Recent evidence indicates that hibernation phenology in *M. nattereri* is shifting rapidly in response to climate change, with adult males reducing hibernation duration by approximately 2.3 days per year in correlation with warming autumn temperatures ( Krivek *et al.*, 2025).

The species produces distinctive social vocalisations during swarming, with call structure varying between maternity colonies and autumn swarming sites, suggesting context-dependent communication functions including contact calls and male advertisement calls ( Schmidbauer & Denzinger, 2019). Echolocation behaviour during prey capture involves characteristic terminal buzz sequences, with buzz I ending approximately 47–63 milliseconds before target contact, corresponding to the species’ reaction time ( Melcón *et al.*, 2007).

High-quality genomic resources for *M. nattereri* will provide valuable tools for investigating the molecular basis of cryptic speciation within the species complex, understanding adaptations related to gleaning behaviour and dietary flexibility, and examining population structure and gene flow dynamics mediated by swarming behaviour ( Josić *et al.*, 2024; Salicini *et al.*, 2011). Recent whole-genome shotgun sequencing has revealed ancient divergence (approximately 3.6 million years ago) between cryptic lineages, yet substantial nuclear and mitochondrial introgression persists in secondary contact zones, indicating incomplete reproductive isolation ( Josić *et al.*, 2024). More broadly, bat genomics has illuminated adaptations in immunity, longevity, and viral tolerance that are relevant to understanding host-pathogen dynamics and zoonotic disease ecology ( Garg *et al.*, 2023; Jebb *et al.*, 2020; Morales *et al.*, 2025; Scheben *et al.*, 2023). Reference-quality genomes enable comparative analyses of gene family evolution, positive selection on functional genes, and regulatory elements underlying physiological specialisations ( Jebb *et al.*, 2020; Liu *et al.*, 2024). For *M. nattereri*, the genome sequence presented here will support conservation genetics by identifying population structure, assessing genetic diversity in threatened populations, and informing management strategies for this species, which faces pressures from habitat loss, climate change, and human-wildlife conflict in roosting sites ( Frick *et al.*, 2020; Zeale *et al.*, 2016).

## Methods

### Sample acquisition

The specimen used for genome sequencing was a male *Myotis nattereri* (specimen ID SAN00003377, ToLID mMyoNat1), collected from Kernascléden, Morbihan, Brittany, France (latitude 47.6508, longitude −3.0705) on 2021-08-17. The specimen was collected and identified by Matthieu Ménage and Gwendoline Dumenil. The injured bat died at the rehabilitation centre. Within one hour of death, multiple tissue samples were collected including flight muscle, heart, kidney, spleen, liver, and wing tissue. Samples were placed in tubes without chemical preservative and initially stored at −20 °C. The samples were transferred to University College Dublin (UCD) and stored at −80 °C for long-term preservation. The same specimen was used for RNA sequencing.

### Nucleic acid extraction

Detailed protocols for nucleic acid extraction developed at the Wellcome Sanger Institute (WSI) Tree of Life Core Laboratory are available on protocols.io ( Howard *et al.*, 2025). The mMyoNat1 sample was weighed and triaged to determine the appropriate extraction protocol. Tissue from the heart was homogenised by powermashing using a PowerMasher II tissue disruptor. High molecular weight (HMW) DNA was extracted using the Automated MagAttract v2 protocol. DNA was sheared into an average fragment size of 12–20 kb following the Megaruptor®3 for LI PacBio protocol. Sheared DNA was purified by automated SPRI (solid-phase reversible immobilisation).

The concentration of the sheared and purified DNA was assessed using a Nanodrop spectrophotometer and Qubit Fluorometer using the Qubit dsDNA High Sensitivity Assay kit. Fragment size distribution was evaluated by running the sample on the FemtoPulse system. For this sample, the final post-shearing DNA had a Qubit concentration of 37.8 ng/μL and a yield of 4 914.00 ng.

RNA was extracted from brain tissue of mMyoNat1 in the Tree of Life Laboratory at the WSI using the RNA Extraction: Automated MagMax™ *mir*Vana protocol. The RNA concentration was assessed using a Nanodrop spectrophotometer and a Qubit Fluorometer using the Qubit RNA Broad-Range Assay kit. Analysis of the integrity of the RNA was done using the Agilent RNA 6000 Pico Kit and Eukaryotic Total RNA assay.

### PacBio HiFi library preparation and sequencing

Library preparation and sequencing were performed at the WSI Scientific Operations core. Libraries were prepared using the SMRTbell Prep Kit 3.0 (Pacific Biosciences, California, USA), following the manufacturer’s instructions. The kit includes reagents for end repair/A-tailing, adapter ligation, post-ligation SMRTbell bead clean-up, and nuclease treatment. Size selection and clean-up were performed using diluted AMPure PB beads (Pacific Biosciences). DNA concentration was quantified using a Qubit Fluorometer v4.0 (ThermoFisher Scientific) and the Qubit 1X dsDNA HS assay kit. Final library fragment size was assessed with the Agilent Femto Pulse Automated Pulsed Field CE Instrument (Agilent Technologies) using the gDNA 55 kb BAC analysis kit.

The sample was sequenced on a Revio instrument (Pacific Biosciences). The prepared library was normalised to 2 nM, and 15 μL was used for making complexes. Primers were annealed and polymerases bound to generate circularised complexes, following the manufacturer’s instructions. Complexes were purified using 1.2X SMRTbell beads, then diluted to the Revio loading concentration (200–300 pM) and spiked with a Revio sequencing internal control. The sample was sequenced on a Revio 25 M SMRT cell. The SMRT Link software (Pacific Biosciences), a web-based workflow manager, was used to configure and monitor the run and to carry out primary and secondary data analysis.

### Hi-C

***Sample preparation and crosslinking***

The Hi-C sample was prepared from 20–50 mg of frozen tissue from the heart of the mMyoNat1 sample using the Arima-HiC v2 kit (Arima Genomics). Following the manufacturer’s instructions, tissue was fixed and DNA crosslinked using TC buffer to a final formaldehyde concentration of 2%. The tissue was homogenised using the Diagnocine Power Masher-II. Crosslinked DNA was digested with a restriction enzyme master mix, biotinylated, and ligated. Clean-up was performed with SPRISelect beads before library preparation. DNA concentration was measured with the Qubit Fluorometer (Thermo Fisher Scientific) and Qubit HS Assay Kit. The biotinylation percentage was estimated using the Arima-HiC v2 QC beads.

***Hi-C library preparation and sequencing***

Biotinylated DNA constructs were fragmented using a Covaris E220 sonicator and size selected to 400–600 bp using SPRISelect beads. DNA was enriched with Arima-HiC v2 kit Enrichment beads. End repair, A-tailing, and adapter ligation were carried out with the NEBNext Ultra II DNA Library Prep Kit (New England Biolabs), following a modified protocol where library preparation occurs while DNA remains bound to the Enrichment beads. Library amplification was performed using KAPA HiFi HotStart mix and a custom Unique Dual Index (UDI) barcode set (Integrated DNA Technologies). Depending on sample concentration and biotinylation percentage determined at the crosslinking stage, libraries were amplified with 10–16 PCR cycles. Post-PCR clean-up was performed with SPRISelect beads. Libraries were quantified using the AccuClear Ultra High Sensitivity dsDNA Standards Assay Kit (Biotium) and a FLUOstar Omega plate reader (BMG Labtech).

Prior to sequencing, libraries were normalised to 10 ng/μL. Normalised libraries were quantified again to create equimolar and/or weighted 2.8 nM pools. Pool concentrations were checked using the Agilent 4200 TapeStation (Agilent) with High Sensitivity D500 reagents before sequencing. Sequencing was performed using paired-end 150 bp reads on the Illumina NovaSeq X.

### RNA library preparation and sequencing

Libraries were prepared using the NEBNext® Ultra™ II Directional RNA Library Prep Kit for Illumina (New England Biolabs), following the manufacturer’s instructions. Poly(A) mRNA in the total RNA solution was isolated using oligo (dT) beads, converted to cDNA, and uniquely indexed; 14 PCR cycles were performed. Libraries were size-selected to produce fragments between 100–300 bp. Libraries were quantified, normalised, pooled to a final concentration of 2.8 nM, and diluted to 150 pM for loading. Sequencing was carried out on the Illumina NovaSeq X, generating paired-end reads.

### Genome assembly

Prior to assembly of the PacBio HiFi reads, a database of *k*-mer counts ( *k* = 31) was generated from the filtered reads using FastK. GenomeScope2 ( Ranallo-Benavidez *et al.*, 2020) was used to analyse the *k*-mer frequency distributions, providing estimates of genome size, heterozygosity, and repeat content.

The HiFi reads were assembled using Hifiasm in Hi-C phasing mode ( Cheng *et al.*, 2021, 2022), producing two haplotypes. Hi-C reads ( Rao *et al.*, 2014) were mapped to the primary contigs using bwa-mem2 ( Vasimuddin *et al.*, 2019). Contigs were further scaffolded with Hi-C data in YaHS ( Zhou *et al.*, 2023), using the --break option for handling potential misassemblies. The scaffolded assemblies were evaluated using Gfastats ( Formenti *et al.*, 2022), BUSCO ( Manni *et al.*, 2021) and MerquryFK ( Rhie *et al.*, 2020).

The mitochondrial genome was assembled using MitoHiFi ( Uliano-Silva *et al.*, 2023).

### Assembly curation

The assembly was decontaminated using the Assembly Screen for Cobionts and Contaminants ( ASCC) pipeline. TreeVal was used to generate the flat files and maps for use in curation. Manual curation was conducted primarily in PretextView and HiGlass ( Kerpedjiev *et al.*, 2018). Scaffolds were visually inspected and corrected as described by Howe *et al*. (2021). Manual corrections included 18 breaks and 152 joins. This reduced the scaffold count by 22.2%, increased the scaffold N50 by 6.9%, and increased the total assembly length by 1.1%. The curation process is described at sanger-tol/curation-resources . PretextSnapshot was used to generate a Hi-C contact map of the final assembly.

### Assembly quality assessment

The MerquryFK tool ( Rhie *et al.*, 2020) was run in a Singularity container ( Kurtzer *et al.*, 2017) to evaluate *k*-mer completeness and assembly quality for both haplotypes using the *k*-mer database ( *k* = 31) computed prior to genome assembly. The analysis outputs included assembly QV scores and completeness statistics.

The genome was analysed using the BlobToolKit pipeline, a Nextflow implementation of the earlier Snakemake version ( Challis *et al.*, 2020). PacBio reads were aligned to the assembly using minimap2 ( Li, 2018) and processed with SAMtools ( Danecek *et al.*, 2021) to generate coverage tracks. The pipeline runs BUSCO ( Manni *et al.*, 2021) using lineages identified from the NCBI Taxonomy ( Schoch *et al.*, 2020). For the three basal BUSCO lineages, eukaryota, bacteria and archaea, BUSCO genes are aligned to the UniProt Reference Proteomes database ( Bateman *et al.*, 2023) using DIAMOND BLASTp ( Buchfink *et al.*, 2021). The genome assembly is divided into chunks based on the density of BUSCO genes from the closest taxonomic lineage, and each chunk is aligned to the UniProt Reference Proteomes database using DIAMOND BLASTx. Sequences without DIAMOND BLASTx hits are extracted using seqtk and aligned to the NT database using BLASTn ( Altschul *et al.*, 1990). The outputs are consolidated into a BlobDir for visualisation in BlobToolKit. The BlobToolKit pipeline was developed using nf-core tooling ( Ewels *et al.*, 2020) and MultiQC ( Ewels *et al.*, 2016), with containerisation through Docker ( Merkel, 2014) and Singularity ( Kurtzer *et al.*, 2017).

## Genome sequence report

### Sequence data

**Figure 2.**
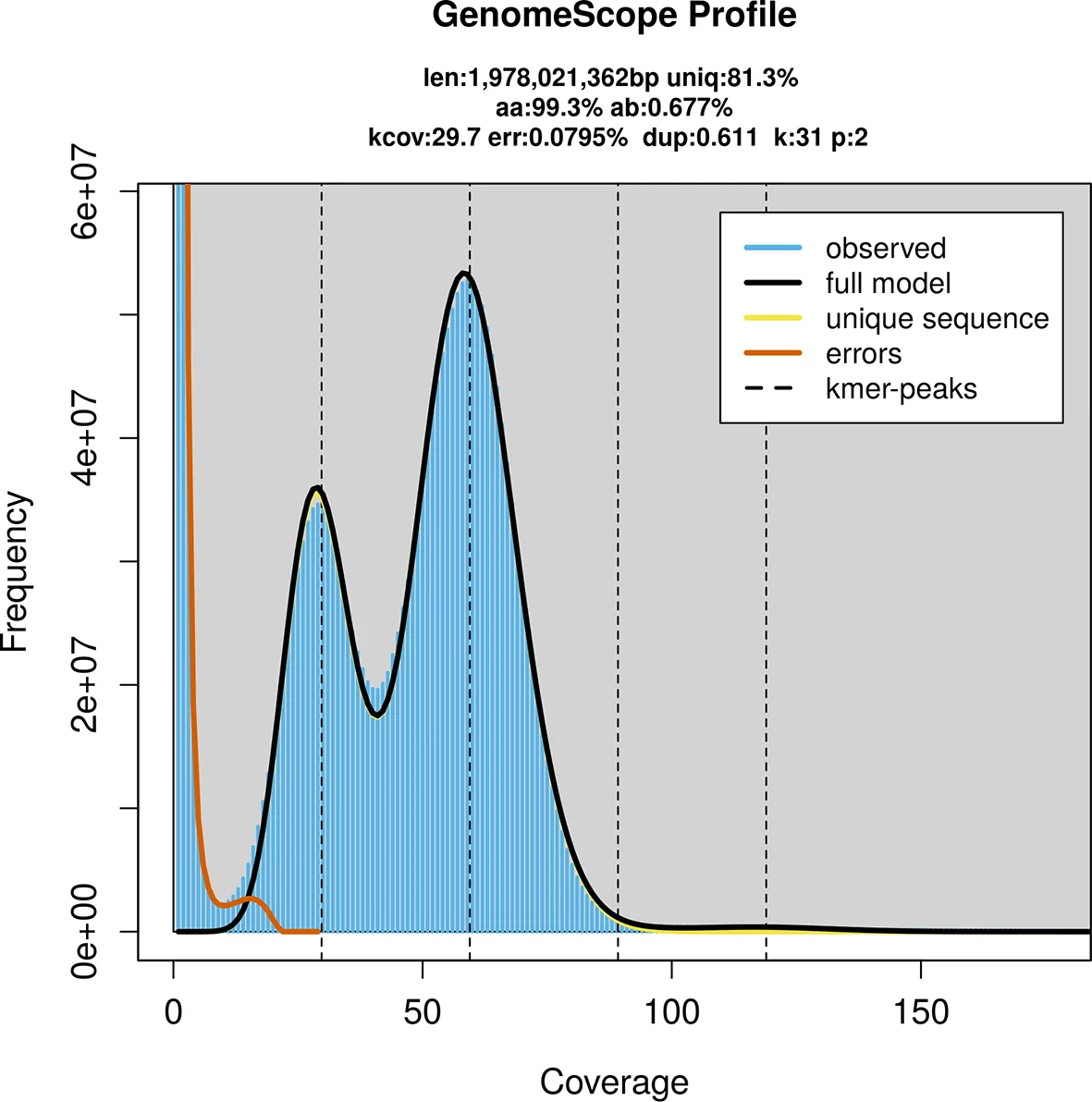
Frequency distribution of *k*-mers generated using GenomeScope2. The plot shows observed and modelled *k*-mer spectra, providing estimates of genome size, heterozygosity, and repeat content based on unassembled sequencing reads.

PacBio sequencing of the *Myotis nattereri* specimen generated 120.99 Gb (gigabases) from 13.62 million reads, which were used to assemble the genome. GenomeScope2.0 analysis estimated the haploid genome size at 1 978.02 Mb, with a heterozygosity of 0.68% and repeat content of 18.77% ( Figure 2). These estimates guided expectations for the assembly. Based on the estimated genome size, the sequencing data provided approximately 59× coverage. Hi-C sequencing produced 284.78 Gb from 942.97 million reads, which were used to scaffold the assembly. RNA sequencing data were also generated and are available in public sequence repositories. Table 1 summarises the specimen and sequencing details.

**Table 1.** Specimen and sequencing data for *Myotis nattereri* (BioProject PRJEB77440).

Platform	PacBio HiFi	Hi-C	RNA-seq
**ToLID**	mMyoNat1	mMyoNat1	mMyoNat1
**Specimen ID**	SAN00003377	SAN00003377	SAN00003377
**BioSample (source individual)**	SAMEA114614248	SAMEA114614248	SAMEA114614248
**BioSample (tissue)**	SAMEA114614262	SAMEA114614262	SAMEA114614250
**Tissue**	heart	heart	brain
**Instrument**	Revio	Illumina NovaSeq X	Illumina NovaSeq X
**Run accessions**	ERR13362626; ERR13362625; ERR14749947	ERR13363410	ERR13493971
**Read count total**	13.62 million	942.97 million	37.42 million
**Base count total**	120.99 Gb	284.78 Gb	11.30 Gb

### Assembly statistics

The genome was assembled into two haplotypes using Hi-C phasing. Haplotype 1 was curated to chromosome level, while haplotype 2 was assembled to scaffold level. The final assembly has a total length of 2 138.58 Mb in 211 scaffolds, with 1 620 gaps, and a scaffold N50 of 100.0 Mb ( Table 2).

**Table 2.** Genome assembly statistics for *Myotis nattereri.*

Genome assembly	Haplotype 1	Haplotype 2
**Assembly name**	mMyoNat1.hap1.2	mMyoNat1.hap2.2
**Assembly accession**	GCA_964212035.2	GCA_964212025.2
**Assembly level**	chromosome	scaffold
**Span (Mb)**	2 138.58	1 932.61
**Number of chromosomes**	23	-
**Number of contigs**	436	309
**Contig N50**	44.23 Mb	47.44 Mb
**Number of scaffolds**	211	173
**Scaffold N50**	100.0 Mb	99.19 Mb
**Longest scaffold length (Mb)**	231.24	-
**Sex chromosomes**	X and Y	-
**Organelles**	Mitochondrial genome: 17.27 kb	-

**Figure 3.**
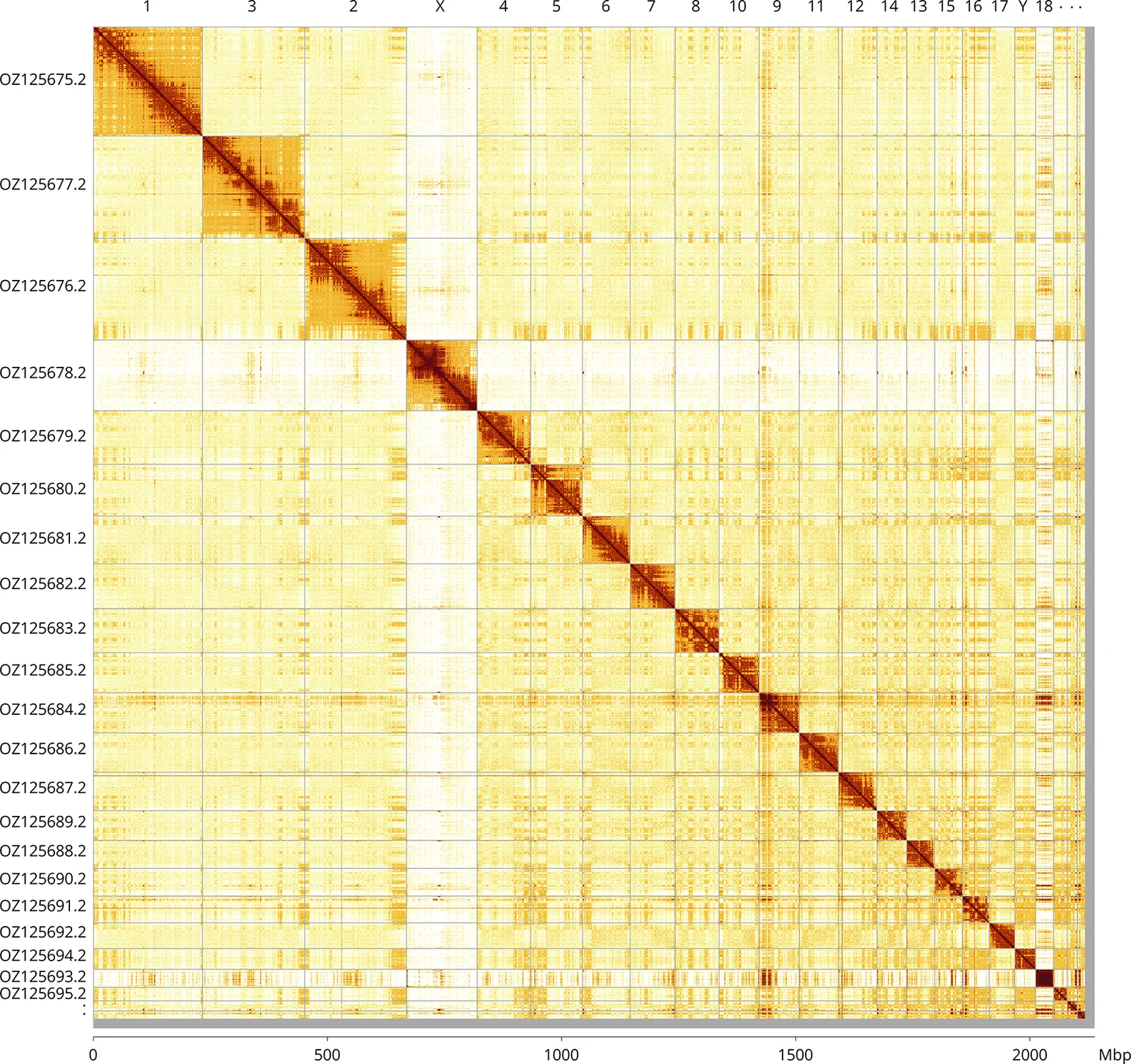
Hi-C contact map of the *Myotis nattereri* genome assembly. Assembled chromosomes are shown in order of size and labelled along the axes, with a megabase scale shown below. The plot was generated using PretextSnapshot.

Most of the haplotype 1 assembly sequence (98.98%) was assigned to 23 chromosomal-level scaffolds, representing 21 autosomes and the X and Y sex chromosomes. These chromosome-level scaffolds, confirmed by Hi-C data, are named according to size ( Figure 3; Table 3). During curation, we observed that contigs on the Y chromosome from 4.8–31.4 Mb have uncertain order and orientation.

**Table 3.** Chromosomal pseudomolecules in the haplotype 1 genome assembly of *Myotis nattereri* mMyoNat1.

INSDC accession	Molecule	Length (Mb)	GC%
OZ125675.2	1	231.24	42
OZ125676.2	2	216.31	41.50
OZ125677.2	3	216.57	42.50
OZ125679.2	4	115.24	42.50
OZ125680.2	5	110.62	44
OZ125681.2	6	100	41.50
OZ125682.2	7	95.22	41.50
OZ125683.2	8	94.65	44
OZ125684.2	9	83.86	44
OZ125685.2	10	84.18	44.50
OZ125686.2	11	83.64	42
OZ125687.2	12	83.17	43.50
OZ125688.2	13	61.90	44.50
OZ125689.2	14	61.95	44
OZ125690.2	15	58	47
OZ125691.2	16	56.82	47.50
OZ125692.2	17	55.14	42.50
OZ125693.2	18	43.50	46.50
OZ125695.2	19	28.98	49.50
OZ125696.2	20	17	48
OZ125697.2	21	18.43	48.50
OZ125678.2	X	154.65	41
OZ125694.2	Y	45.63	44.50

The mitochondrial genome was also assembled (length 17.27 kb, OZ125698.2). This sequence is included as a contig in the multifasta file of the genome submission and as a standalone record.

### Assembly quality metrics

**Figure 4.**
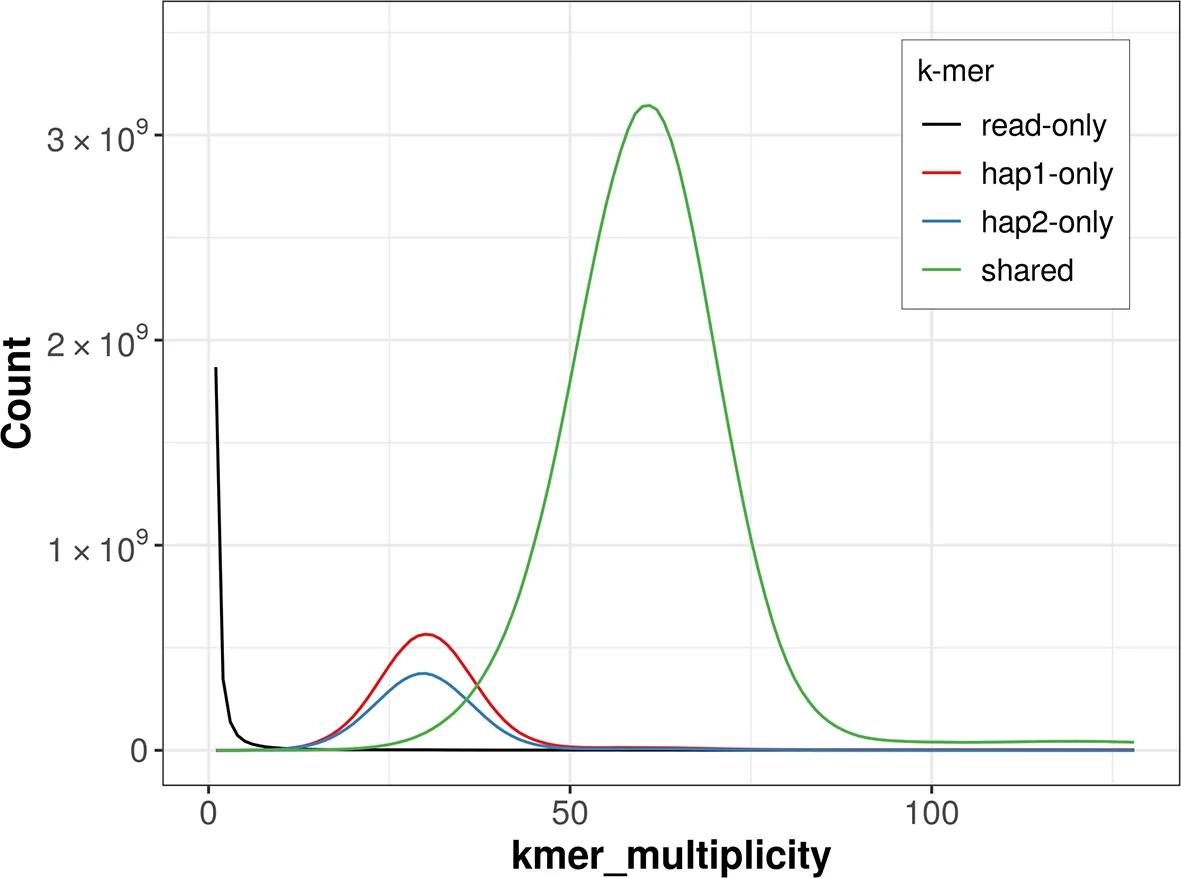
Evaluation of *k*-mer completeness using MerquryFK. This plot illustrates the recovery of *k*-mers from the original read data in the final assemblies. The horizontal axis represents *k*-mer multiplicity, and the vertical axis shows the number of *k*-mers. The black curve represents *k*-mers that appear in the reads but are not assembled. The green curve corresponds to *k*-mers shared by both haplotypes, and the red and blue curves show *k*-mers found only in one of the haplotypes.

For haplotype 1, the estimated QV is 62.5, and for haplotype 2, 62.0. When the two haplotypes are combined, the assembly achieves an estimated QV of 62.2. The *k*-mer completeness is 88.31% for haplotype 1, 82.92% for haplotype 2, and 99.70% for the combined haplotypes ( Figure 4).

**Figure 5.**
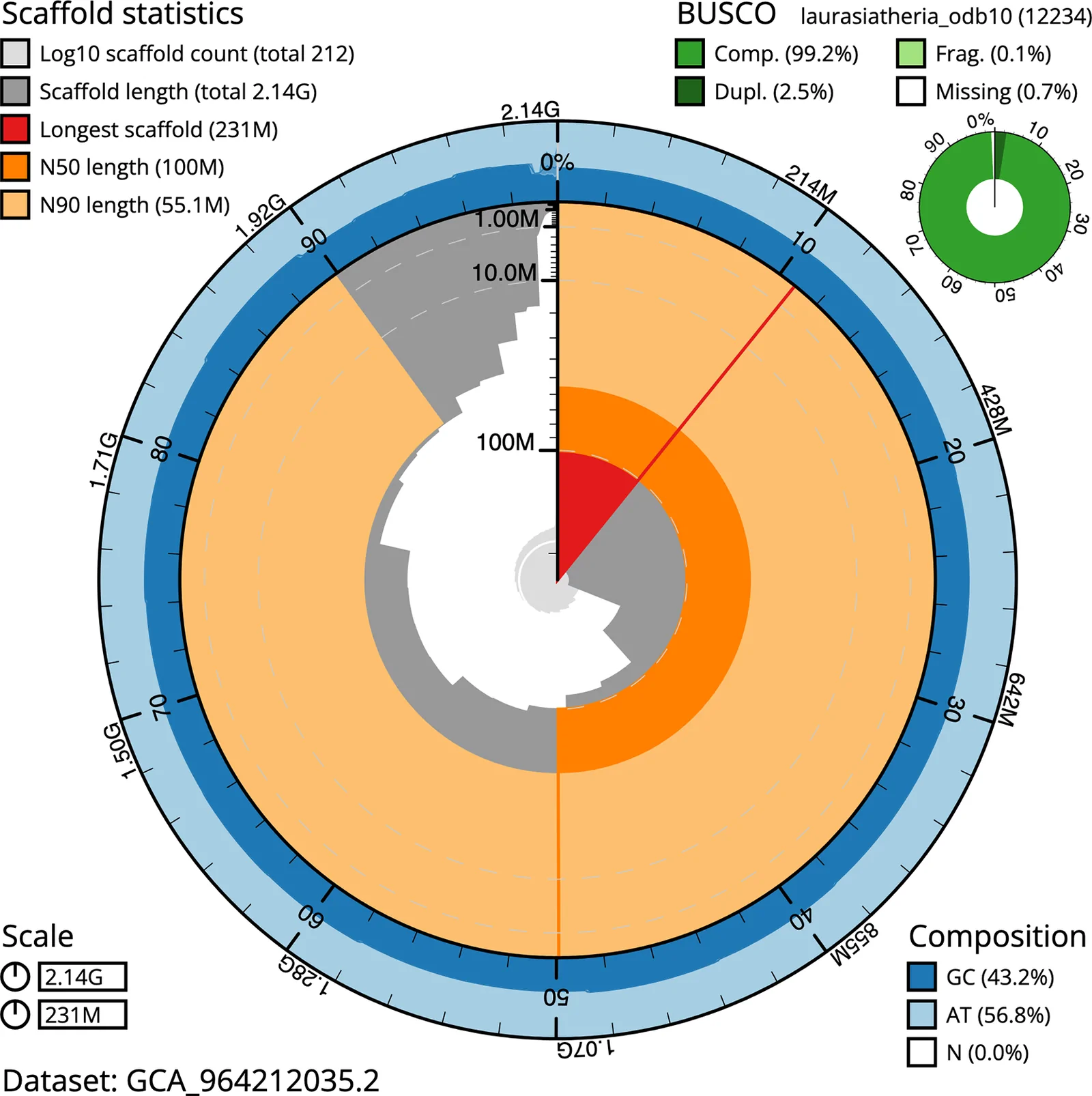
Assembly metrics for mMyoNat1.hap1.2. The BlobToolKit snail plot provides an overview of assembly metrics and BUSCO gene completeness. The circumference represents the length of the whole genome sequence, and the main plot is divided into 1 000 bins around the circumference. The outermost blue tracks display the distribution of GC, AT, and N percentages across the bins. Scaffolds are arranged clockwise from longest to shortest and are depicted in dark grey. The longest scaffold is indicated by the red arc, and the deeper orange and pale orange arcs represent the N50 and N90 lengths. A light grey spiral at the centre shows the cumulative scaffold count on a logarithmic scale. A summary of complete, fragmented, duplicated, and missing BUSCO genes in the set is presented at the top right. An interactive version of this figure can be accessed on the BlobToolKit viewer.

**Figure 6.**
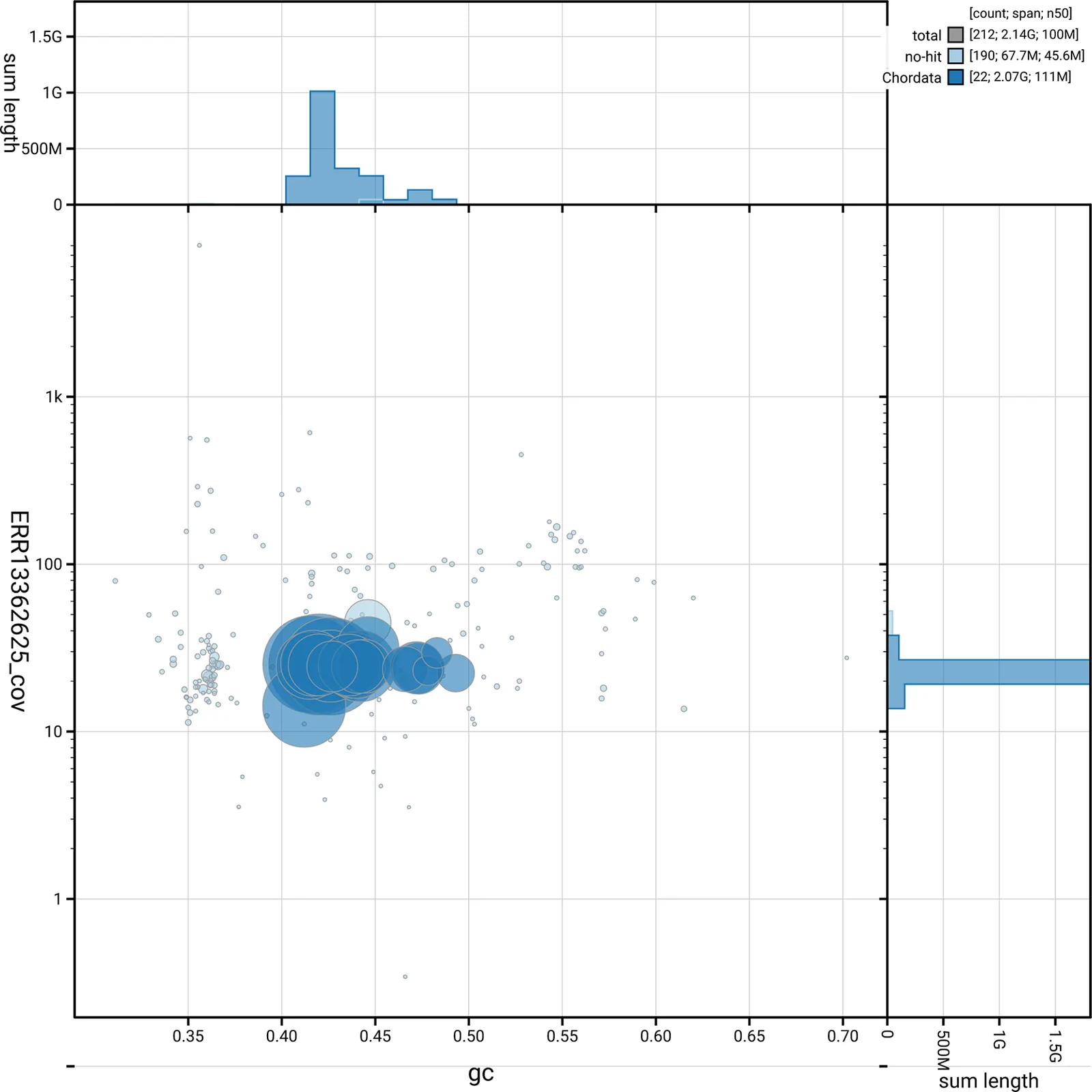
BlobToolKit blob plot for mMyoNat1.hap1.2. The plot shows base coverage (vertical axis) and GC content (horizontal axis). The circles represent scaffolds, with the size proportional to scaffold length and the colour representing phylum membership. The histograms along the axes display the total length of sequences distributed across different levels of coverage and GC content. An interactive version of this figure is available on the BlobToolKit viewer.

BUSCO analysis using the laurasiatheria_odb10 reference set ( *n* = 12 234) identified 99.2% of the expected gene set (single = 96.8%, duplicated = 2.5%) in haplotype 1. For haplotype 2, BUSCO v.6.0.0 analysis identified 96.6% of the expected gene set (single = 94.5%, duplicated = 2.2%). The snail plot in Figure 5 summarises the scaffold length distribution and other assembly statistics for haplotype 1. The blob plot in Figure 6 shows the distribution of scaffolds by GC proportion and coverage for haplotype 1.

Table 4 lists the assembly metric benchmarks adapted from Rhie *et al.* (2021) and the Earth BioGenome Project Report on Assembly Standards January 2026. The EBP metric, calculated for the haplotype 1, is **7.C.Q62**, meeting the recommended reference standard.

**Table 4.** Earth Biogenome Project summary metrics for the *Myotis nattereri* assembly.

Measure	Value	Benchmark
EBP summary (haplotype 1)	7.C.Q62	6.C.Q40
Contig N50 length	44.23 Mb	≥ 1 Mb
Scaffold N50 length	100 Mb	= chromosome N50
Consensus quality (QV)	Haplotype 1: 62.5; haplotype 2: 62.0; combined: 62.2	≥ 40
*k*-mer completeness	Haplotype 1: 88.31%; Haplotype 2: 82.92%; combined: 99.70%	≥ 95%
BUSCO	C:99.2% [S:96.8%, D:2.5%], F:0.1%, M:0.7%, n:12 234	S > 90%; D < 5%
Percentage of assembly assigned to chromosomes	98.98%	≥ 90%

**Notes:** The EBP summary uses log10(Contig N50); chromosome-level (C) or log10(Scaffold N50); Q (Merqury QV). BUSCO: C = complete; S = single-copy; D = duplicated; F = fragmented; M = missing; n = orthologues

**Table 5.** Software versions and sources used for *Myotis nattereri.*

Software	Version	Source
BLAST	2.14.0	ftp://ftp.ncbi.nlm.nih.gov/blast/executables/blast+/
BlobToolKit	4.4.6	https://github.com/blobtoolkit/blobtoolkit
BUSCO	6.0.0	https://gitlab.com/ezlab/busco
bwa-mem2	2.2.1	https://github.com/bwa-mem2/bwa-mem2
DIAMOND	2.1.8	https://github.com/bbuchfink/diamond
fasta_windows	0.2.4	https://github.com/tolkit/fasta_windows
FastK	1.1	https://github.com/thegenemyers/FASTK
GenomeScope2.0	2.0.1	https://github.com/tbenavi1/genomescope2.0
Gfastats	1.3.6	https://github.com/vgl-hub/gfastats
Hifiasm	0.19.8-r603	https://github.com/chhylp123/hifiasm
HiGlass	1.13.4	https://github.com/higlass/higlass
MerquryFK	1.1.2	https://github.com/thegenemyers/MERQURY.FK
Minimap2	2.28-r1209	https://github.com/lh3/minimap2
MitoHiFi	3	https://github.com/marcelauliano/MitoHiFi
MultiQC	1.14; 1.17 and 1.18	https://github.com/MultiQC/MultiQC
Nextflow	24.10.4	https://github.com/nextflow-io/nextflow
PretextSnapshot	0.0.5	https://github.com/sanger-tol/PretextSnapshot
PretextView	1.0.3	https://github.com/sanger-tol/PretextView
samtools	1.21	https://github.com/samtools/samtools
sanger-tol/ascc	0.1.0	https://github.com/sanger-tol/ascc
sanger-tol/blobtoolkit	v0.9.0	https://github.com/sanger-tol/blobtoolkit
sanger-tol/curationpretext	1.4.2	https://github.com/sanger-tol/curationpretext
Seqtk	1.3	https://github.com/lh3/seqtk
Singularity	3.9.0	https://github.com/sylabs/singularity
TreeVal	1.4.0	https://github.com/sanger-tol/treeval
YaHS	1.2a.2	https://github.com/c-zhou/yahs

### Genome annotation report

The *Myotis nattereri* genome assembly (GCA_964212035.2) was annotated by Ensembl at the European Bioinformatics Institute (EBI). This annotation includes 45 448 transcribed mRNAs from 19 245 protein-coding and 13 000 non-coding genes. The average transcript length is 36 587.13 bp, with an average of 1.39 coding transcripts per gene and 8.10 exons per transcript. Further details of this annotation are available from the Ensembl annotation page.

## Author information

Contributors are listed at the following links:

• Members of the Wellcome Sanger Institute Tree of Life Management, Samples and Laboratory team
• Members of Wellcome Sanger Institute Scientific Operations – Sequencing Operations
• Members of the Wellcome Sanger Institute Tree of Life Core Informatics team
• Members of the Tree of Life Core Informatics collective
• Members of the Darwin Tree of Life Consortium

## Wellcome Sanger Institute – Legal and Governance

The materials that have contributed to this genome note have been supplied by a Darwin Tree of Life Partner. The submission of materials by a Darwin Tree of Life Partner is subject to the **‘Darwin Tree of Life Project Sampling Code of Practice’**, which can be found in full on the Darwin Tree of Life website. By agreeing with and signing up to the Sampling Code of Practice, the Darwin Tree of Life Partner agrees they will meet the legal and ethical requirements and standards set out within this document in respect of all samples acquired for, and supplied to, the Darwin Tree of Life Project. Further, the Wellcome Sanger Institute employs a process whereby due diligence is carried out proportionate to the nature of the materials themselves, and the circumstances under which they have been/are to be collected and provided for use. The purpose of this is to address and mitigate any potential legal and/or ethical implications of receipt and use of the materials as part of the research project, and to ensure that in doing so we align with best practice wherever possible. The overarching areas of consideration are:

• Ethical review of provenance and sourcing of the material
• Legality of collection, transfer and use (national and international)

Each transfer of samples is further undertaken according to a Research Collaboration Agreement or Material Transfer Agreement entered into by the Darwin Tree of Life Partner, Genome Research Limited (operating as the Wellcome Sanger Institute), and in some circumstances, other Darwin Tree of Life collaborators.

## Data Availability

European Nucleotide Archive: Myotis nattereri (Natterer’s bat). Accession number PRJEB77440. The genome sequence is released openly for reuse. The *Myotis nattereri* genome sequencing initiative is part of the Darwin Tree of Life Project (PRJEB40665), the Sanger Institute Tree of Life Programme (PRJEB43745), the Vertebrate Genomes Project (PRJNA489243) and Bat1K (PRJNA489245). All raw sequence data and the assembly have been deposited in INSDC databases. Raw data and assembly accession identifiers are reported in Tables 1 and 2. Production code used in genome assembly at the WSI Tree of Life is available at https://github.com/sanger-tol . Table 5 lists software versions used in this study.
