# Supplementary material for: The genome sequence of Natterer’s Bat, Myotis nattereri (Kuhl, 1817) (Chiroptera: Vespertilionidae)
Source: Wellcome Open Res. 2026 Jul 25;11:466. [Version 1] doi: 10.12688/wellcomeopenres.27171.1 (PMC13560790; doi:10.12688/wellcomeopenres.27171.1)
Supplement: Peer review history

# Reviewer report: Reviewer response for version 1

**DOI:** 10.21956/wellcomeopenres.29910.r163365

**Published:** 2026-08-11

**Authors:** Inge Seim

**Affiliations:** 1 Institute of Deep-Sea Science and Engineering, Sanya, China

In their Data Note, Ménage and colleagues generated a high-quality genome of *Myotis nattereri*. From memory, genus *Myotis* comprises about 10% of bats, meaning that this assembly is certainly warranted. Sex chromosomes were also assembled, although the chromosome Y assembly had some issues—as to be expected from an assembly not employing ultralong ONT or similar methods—but this is not a critique. I also appreciate that the annotated assembly is already available on Ensembl. **I have no major concerns and recommend acceptance of this genome report**.

 I have a few comments for the authors that they may choose to take into consideration:

 • It may be useful to mention how many *Myotis* bats have been sequenced (including at what relative “quality” level), and that the genus is speciose.

 • Please remove trademark and copyright symbols (see RNA library preparation section).

 • I would mention in the Results that the RNA-seq was from brain-only. This is, of course, a great tissue for annotation given its complexity, but if one says “RNA sequencing data were also generated and are available in public sequence repositories”, it suggests the typical trifecta of brain+liver+kidney RNA-seq. Just add "brain" in front of "RNA sequencing data ...".

 • Table 1: it is a bit misleading to list the mitochondrial genome under “Haplotype 1”. This confuses me. I assume MitoHiFi outputs a single representative sequence from whatever long MT-reads that are not NUMTs are present in the PacBio HiFi reads?

 • Table 2: Show significant figures, e.g., chr1, chr5 GC%?

 • I realise https://beta.ensembl.org/genome/cb1c7d20-7c99-42f7-a6ec-c3c8c1c25d1d contains information on the non-coding genes, but perhaps mention what % of the non-coding genes are assigned as long non-coding (9,195/13,000 = 70.73%) or short non-coding?      

Are sufficient details of methods and materials provided to allow replication by others?

Yes

Is the rationale for creating the dataset(s) clearly described?

Yes

Are the datasets clearly presented in a useable and accessible format?

Yes

Are the protocols appropriate and is the work technically sound?

Yes

Reviewer Expertise:

comparative genomics

I confirm that I have read this submission and believe that I have an appropriate level of expertise to confirm that it is of an acceptable scientific standard.

# Reviewer report: Reviewer response for version 1

**DOI:** 10.21956/wellcomeopenres.29910.r163360

**Published:** 2026-08-10

**Authors:** Bo Wang

**Affiliations:** 1 Duke-NUS Medical School, 8 College Road, Singapore, Singapore

Ménage et al. describe the assembly of a genome sequence from a male Natterer’s bat ( *Myotis nattereri*) collected in France in 2021. The authors characterized the genome and identified 19,245 protein-coding genes. This study has the potential to support conservation genetics by helping to identify population structure, assess genetic diversity, and inform management strategies for this species. Overall, the manuscript is clearly written. I have only a few minor comments for the authors to consider.

1. Background: Among the approximately 120 extant species within the genus *Myotis*, how many bat genomes have been sequenced to date?
2. Methods, Sample acquisition: How did the authors determine that the bat had died within one hour prior to collection? In addition, was ethics approval or an appropriate collection permit obtained for the use of this animal specimen?
3. Table 1: Why were heart tissue samples used for PacBio HiFi and Hi-C sequencing, whereas brain tissue was used for RNA-seq? Could the large difference in total base count be related to the tissue type or sample quality?
4. Figures 3–6: The authors could provide more detailed descriptions of the results shown in these figures, particularly by highlighting the key findings and explaining the significance of each analysis.

Are sufficient details of methods and materials provided to allow replication by others?

Yes

Is the rationale for creating the dataset(s) clearly described?

Yes

Are the datasets clearly presented in a useable and accessible format?

Partly

Are the protocols appropriate and is the work technically sound?

Yes

Reviewer Expertise:

bat virus, evolution, ecology, taxonomy,

I confirm that I have read this submission and believe that I have an appropriate level of expertise to confirm that it is of an acceptable scientific standard.
